# Ant Brood Function as Life Preservers during Floods

**DOI:** 10.1371/journal.pone.0089211

**Published:** 2014-02-19

**Authors:** Jessica Purcell, Amaury Avril, Geoffrey Jaffuel, Sarah Bates, Michel Chapuisat

**Affiliations:** 1 Department of Ecology and Evolution, University of Lausanne, Lausanne, Switzerland; 2 Institute of Biology, University of Neuchâtel, Neuchâtel, Switzerland; Université Paris 13, France

## Abstract

Social organisms can surmount many ecological challenges by working collectively. An impressive example of such collective behavior occurs when ants physically link together into floating ‘rafts’ to escape from flooded habitat. However, raft formation may represent a social dilemma, with some positions posing greater individual risks than others. Here, we investigate the position and function of different colony members, and the costs and benefits of this functional geometry in rafts of the floodplain-dwelling ant *Formica selysi*. By causing groups of ants to raft in the laboratory, we observe that workers are distributed throughout the raft, queens are always in the center, and 100% of brood items are placed on the base. Through a series of experiments, we show that workers and brood are extremely resistant to submersion. Both workers and brood exhibit high survival rates after they have rafted, suggesting that occupying the base of the raft is not as costly as expected. The placement of all brood on the base of one cohesive raft confers several benefits: it preserves colony integrity, takes advantage of brood buoyancy, and increases the proportion of workers that immediately recover after rafting.

## Introduction

Social organisms have an advantage when responding to ecological adversity: they can react in a collective and organized way, working together to perform tasks that a solitary individual could not achieve [1], [2], [3]. For instance, some societies respond to predators by mounting a coordinated defense, as in leaf-cutter ants, which form a defensive line featuring large major workers and teams of smaller workers to block invading army ants [4]. Other species link their bodies together to achieve a mutual goal, as in Japanese honeybees, which will surround large predatory hornets and form an ‘oven,’ raising the interior temperature to kill the intruder [5]. The latter case is an example of a ‘collective structure.’ These self-assembled collective structures can provide defense, shelter, thermoregulation, bridges over obstacles, or a means of transportation [6]. Although collective structures are widespread, particularly in the social Hymenoptera, their functional geometry, defined as the position and function of individuals within the structure, generally remains poorly understood.

In many collective structures, different castes occupy specific positions. In the ‘bivouac’ nests of army ants and in bee or wasp swarms, for example, workers form protective layers around more vulnerable queens and brood [6], [7], [8]. If some positions are safe and others risky, the configuration of these structures suggests that altruism or coercion may be inherent in such self-assemblages. However, the costs and benefits of specific positions are difficult to measure, and the position of each individual may also depend on how its particular physical properties function in the structure.

Ant rafts provide a useful model of a collective structure in which occupancy of some positions – namely positions on the raft base – may be detrimental and thus reflect altruistic self-sacrifice. Alternatively, positions may be filled based on the functional properties of individuals. Many floodplain-dwelling ant species form rafts. Colony members assemble into a floating platform by linking tarsus-tarsus or mandible-tarsus [9], [10]. In the fire ant *Solenopsis invicta*, recent studies have investigated the physical properties of rafts [9], as well as raft formation, longevity, and success rates under controlled conditions [11]. Adams et al. [11] further noted qualitatively that fire ants tended to place larger brood on the raft base, which allowed rafts to remain afloat longer than those consisting of only workers. They speculated that brood may be more buoyant than workers. The finding that rafting ants place some brood on the raft base raises the question of whether this action imposes costs on the brood and/or benefits the group.

**Figure 1:**
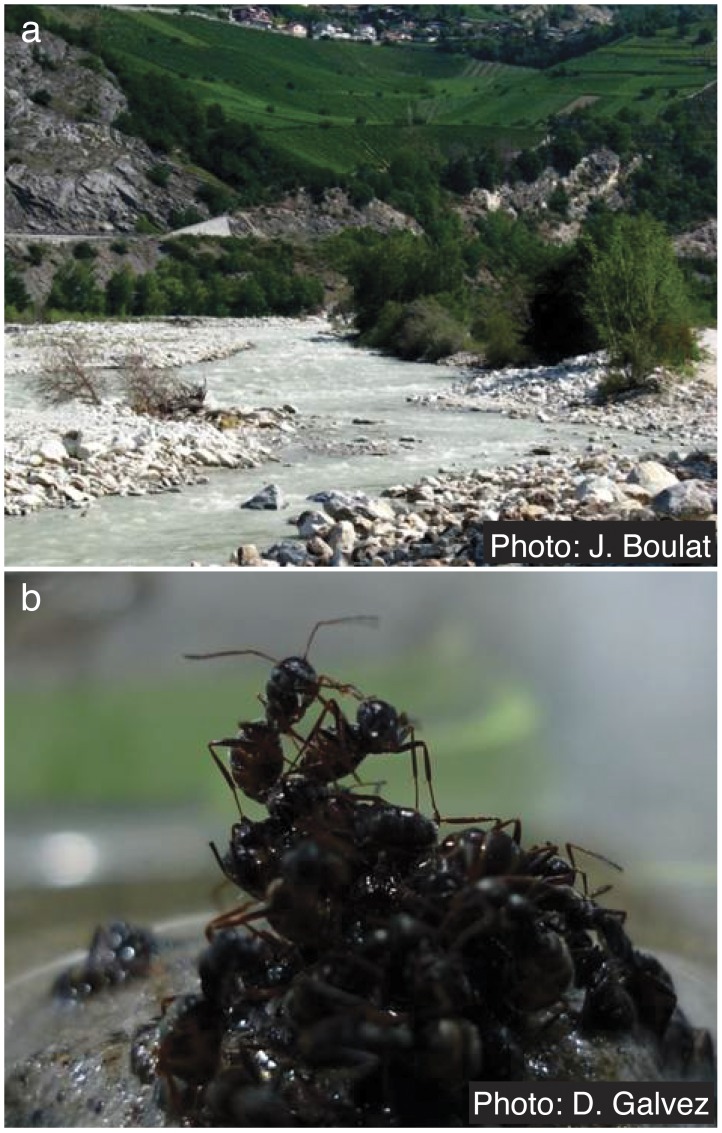
Photos of floodplain habitat in Valais, Switzerland (a) and incipient raft during self-assembly (b).

Here, we investigate the functional geometry of rafts in the ant *Formica selysi*. These ants are abundant in floodplains throughout the Alps and the Pyrenees (Fig. 1a), where floods can cause severe erosion and may submerge nests for days [12]. During floods, colonies have been observed to evacuate their nests and raft to safety (Fig. 1b) [13]. We elicited rafting behavior in the laboratory to investigate where workers, brood, and queens are positioned in the raft, and to what degree their respective positions require altruistic self-sacrifice, and/or reflect functional differences in their physical properties. In a series of experiments, we quantify for the first time the costs and benefits associated with the position of workers and brood in the rafts, and we measure their respective buoyancy. We expect workers to protect the most vulnerable and valuable nest-mates by placing them in the center of the raft, but also to take advantage of the physical properties of each caste to build a robust and buoyant raft.

## Materials and Methods

In 2011 and 2012, we collected workers and brood from field colonies in a large *F. selysi* population along the Rhône River in Valais, Switzerland (7°36′30″E, 46°18′30″N, altitude 565 m). We additionally collected one to two mature queens from each of five polygynous colonies. No specific permit was required to collect this ant species, which is not endangered or protected. Each individual was used in only a single experiment or trial in this study. This population has been monitored for over ten years [14], and large floods causing erosion and nest destruction have been observed during that time (Fig. S1 in File S1) [12]. Flooding of the alluvial plain habitat generally occurs in the late spring through late summer, when brood is present in *F. selysi* colonies [12].

We constructed an apparatus to film raft formation from above and from below simultaneously (Fig. S2 in File S1). To induce rafting, we placed ants on a platform and raised the water level slowly. Initially, we investigated the placement of queens, sexual brood, and worker brood in rafts (Table 1). We then performed a series of experiments to better understand the geometry, function, costs and benefits of ant raft assemblages. Additional details of the rafting apparatus, study species, and methodology are provided in the supporting information.

**Table 1:** Summary of results from rafting experiments.

	group composition	trials	raftduration	positions	comparison
Queen	1 queen	2	30 min.	Queens in center of raft, workers above,on base, and on sides of raft.	
position	2 queens	3		25–50% of workers in contact with the water.	
Sexual brood position	5 brood	5	30 min.	Sexual brood on raft base, workersthroughout but few on base.	
Worker brood position	10 brood	5	30 min.	Worker brood on raft base, workersthroughout but few on base.	
Raftingtolerance	–	10	3 hrs.	Brood on base if present, workersthroughout the raft.	Survival of brood that rafted *versus* brood provided to worker groups after rafting:
of brood	10 brood	10		More workers in contact with waterin the absence of brood.	83% *versus* 79%, paired t-test t_9_ = 0.74, *p* = 0.48
Buoyantmaterialschoice	10 brood +10 wood cylinders	10	30 min.	Brood on base. In some cases, woodcylinders passively included on theperipheral base of the raft, workersthroughout but few on base	Mean numbers of brood items *versus* wood cylinders collected: 9.8±0.2 (standard error) *versus* 1.1±0.5 and incorporated in the raft: 9.8±0.2 *versus* 3.8±0.6, paired Wilcoxon tests V = 55, df = 9, p = 0.0055
Raft	–	10	3 hrs.	Brood on base if present, workersthroughout the raft.	Mean time to disassemble rafts with brood *versus* without brood: 326±37 seconds ± standard error *versus* 230±29 seconds, paired t-test t_9_ = 1.60, p = 0.14;
recovery	10 brood	10		More workers in contact with water inthe absence of brood.	Mean number of unresponsive workers after rafting with brood *versus* without brood: 0.6±0.2 *versus* 3±0.8, paired t-test t_9_ = 3.09, p = 0.013

Each trial involved 60 workers, and each group of workers (and brood) rafted only once. The same groups of workers and brood were used for the raft recovery and rafting tolerance of brood experiments: after the initial raft trials, we observed the raft recovery and later monitored brood eclosion.

### Colony Member Positions

We formed groups of 60 workers collected from each of 15 field colonies, to which we added additional individuals from the same field colonies to constitute three different experimental conditions. We added: (i) one or two queens (N  = 2 and N  = 3, respectively), (ii) ten worker pupae (N  = 5), or (iii) five sexual pupae (N  = 5). Each group was then subjected to a flood, causing them to raft for 30 minutes.

### Submersion Tolerance of Workers

We submerged three workers from each of 14 field colonies, and investigated their resistance to staying underwater. The experimental apparatus consisted of a glass tube that we placed in a large water container, ensuring that no bubbles remained in the tube. We then placed workers individually in the glass tube, so that ants were not able to float to the surface. Following an eight hour submersion, we removed workers to a filter-paper lined box and measured their survival and recovery time.

### Rafting Tolerance of Brood

For each of ten field colonies, we formed two experimental groups of 60 workers and allowed them to raft for three hours; for each colony, one of the groups had ten nest-mate brood items during rafting, while the other group received ten nest-mate brood items after rafting (Table 1). We used a combination of pupae and larvae during this experiment, but found no difference in survival between the two (Binomial test *p*  = 0.51), so we combined them in our subsequent analyses. After rafting, the experimental groups (each with 60 workers and 10 brood items) were placed in boxes (15×13×6 cm) containing one plaster nest and *ad libitum* access to standard ant food and water. The groups were monitored at least five times per week until all brood had either eclosed to adulthood or died. We used a paired t-test to investigate whether brood that experienced rafting exhibited a different survival rate than brood that did not experience rafting.

### Buoyancy of Workers and Brood

We placed individual workers, larvae and pupae from each of eight field colonies in solutions with increasing concentrations of detergent for two minutes and recorded whether they remained afloat (Table S1 in File S1). Detergent decreases the surface tension of water, which reduces buoyancy caused by air trapped on the hydrophobic body surface [9].

### Buoyant Materials Choice Experiment

To test whether workers prefer brood over other buoyant material to form a raft base, we provided them with both brood items and pieces of wood of similar dimensions and weight as the brood (Table 1). We collected workers and brood from 10 field colonies to form 10 replicates. We let groups of 60 workers settle on the watch glass, and placed 10 pupae and 10 wood cylinders at equal distances from the largest group of workers. We then measured the number of pupae and wood cylinders that were actively collected and the number incorporated into the raft (either actively or passively) during 30 minutes of rafting, and compared these measures using paired Wilcoxon tests.

### Raft Recovery

We compared the recovery time of workers from rafts with brood to those from rafts without brood. For each of ten field colonies, we formed two experimental groups: one with 60 workers and the other with 60 workers plus 10 brood items. We filmed the behavior of groups for one hour after three hours of rafting and used paired t-tests to compare the time to disassemble the raft and the number of unresponsive workers.

## Results

### Raft Formation

When only workers are present, rafts are initiated by a single group of workers (about 60–80% of the 60 individuals in our trials) that remain close to one another and begin to form a pile consisting of 2–3 layers of workers as the water level rises. The remaining workers walk between the water’s edge and the group, or engage in trophallaxis, self-grooming or allo-grooming away from the group. These individuals either climb onto the pile or join the outer edge of the aggregation when the water level rises to the raft level. The picture is similar when queens or brood are present. Workers quickly and actively collect brood items from the platform, place them in a single pile and aggregate on top of them. The brood is often repositioned during this phase. As the water level rises, and early in the raft assembly process, queens gradually move to occupy the center of the pile of workers. Brood are held in the mandibles of the workers and maintained on the base of the pile. As above, some workers remain mobile until the water level reaches the raft when queens or brood are present. When they begin to float, rafts have 3–4 layers of workers. *Formica selysi* was reluctant to raft, both in the field [13] and in the laboratory (see supporting information).

### Raft Geometry

Adult queens always occupied the center of the raft (Table 1). The placement of queens ensured that they were neither touching the water nor exposed from above. In contrast, workers systematically placed all sexual and worker-destined brood on the base of the raft (Table 1; see Movie S1, S2). When brood items were present, very few workers occupied a position on the raft base, but without brood, 25–50% of workers had at least partial contact with water.

### Costs of Submersion and Rafting

The cost of rafting was lower than expected, because both workers and brood were highly resistant to submersion in water. After spending eight hours completely under water, 79% of *F. selysi* workers recovered. On average, workers began to move 66±3 minutes (mean ± SE) and began to walk 77±4 minutes after removal from water. Given that workers in rafts usually were not completely submerged, rafting may cause little or no direct mortality to workers, even when they occupy the raft base. However, workers on the raft base need a significant period of time to recover after rafting (see ‘Benefits of raft geometry’ section). Similarly, brood that spent 3 hours on a raft base did not appear to pay a significant cost; brood that rafted survived until eclosion at the same rate as those that did not (Table 1).

### Function of Raft Geometry

Larvae and pupae (with and without cocoons) were significantly more buoyant than workers (Table S1 in File S1), which most likely explains why workers place brood on the raft base. Workers preferred to use brood over wood cylinders, which are also highly buoyant (Table 1, Table S1 in File S1). Wood cylinders were sometimes incorporated into the raft when they were encountered after raft formation.

### Benefits of Raft Geometry

**Figure 2:**
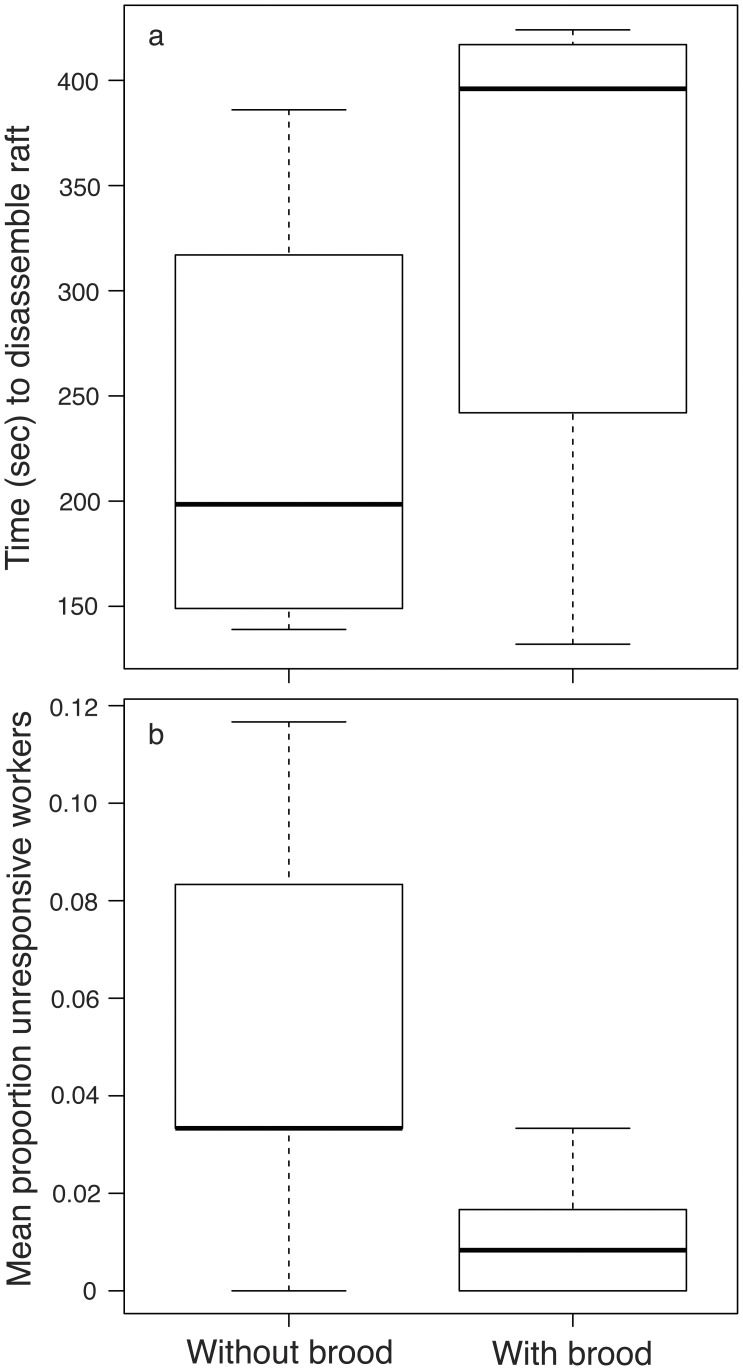
Comparison of recovery of rafts with and without brood: time to disassemble raft (a) and proportion of unresponsive workers after 60 minute recovery period (b).

After rafting, the workers released each other and began to move away from the aggregation. The rafts disassembled with workers from the top and sides of the raft departing first. Brood and unresponsive workers were generally moved to a dry location within 20 minutes and groomed extensively. Rafts with brood tended to take more time to disassemble than rafts without brood, but the difference was not significant (Table 1; Fig. 2). On the other hand, rafts composed of workers and brood had significantly fewer unresponsive workers than those with workers alone (Table 1; Fig. 2).

## Discussion

Some ants have evolved a remarkable ability to self-assemble into rafts in response to floods. The formation of rafts is a progressive and coordinated process, resulting in a collective structure with a well-defined geometry. Strikingly, workers place brood on the base of the raft and use them as a floating platform.

We expected *F. selysi* ants to protect particularly vulnerable or valuable members of their society by placing them in the center of their rafts. Indeed, queens consistently occupied rafts centers, out of the water and protected by workers on all sides. In contrast, ants placed larvae and pupae, both worker and sexual, on the raft base. This geometry did not result from constraints due to lack of workers; rafts generally consisted of three or four layers of workers, so placing brood in an internal position would have been possible if the workers holding brood in their mandibles occupied a higher layer.

We observed little mortality in our experiments, suggesting that the social dilemma facing rafting ants may have less severe consequences than we initially predicted. Contrary to our expectations, even workers and brood that stayed underwater for hours on the raft base exhibited very high survival rates. The reluctance of ants to raft combined with the protective placement of queens in the raft center, however, suggest that there may be other costs or dangers not accounted for in our experiments. Obvious costs of rafting include the risk of losing the nest, of colony fragmentation, and of being washed away to unsuitable habitat. Moreover, predation by fish or exposure to turbulent waters may cause higher mortality than measured in laboratory conditions. Consistent with the hypothesis of elevated risks, fire ants increase the venom in their stings while rafting [15]. Finally, there are likely to be physiological costs associated with submersion in water, including oxygen deprivation, increased CO_2_ levels, and possible thermal effects from cold water.

The collection and placement of brood on the raft base may serve multiple functions. First, brood items are more buoyant than adult workers, and thus serve as flotation devices. When submerged for an extended time, *F. selysi* workers become immobile, and require an hour or more to recover. Thus, workers from rafts with brood recover more quickly, on average, than workers from rafts without brood. This would likely be highly advantageous in the natural environment, where groups need to find cover quickly after reaching shore. Along the same lines, Adams et al. [11] showed that *S. invicta* rafts are able to remain afloat longer when brood items are integrated.

Other essential functions of self-assembling into a single raft are to preserve the progeny, and to keep the colony together [16], [17]. Given that the brood suffers little or no mortality and workers preferentially incorporate brood into the raft over other buoyant materials, we suggest that brood rescue and colony cohesion are the primary motivations to incorporate brood in the raft, while their buoyant properties explain their placement on the base.

The rafts in our study contained fewer ants and brood items than most natural colonies (see supporting information), but given the consistent and deliberate placement of brood and queens across our tests, we expect the functional geometry of rafts to scale up to full size colonies. Other measurements, such as mortality rate and raft recovery, may not scale linearly with raft size and the time spent rafting. Future tests investigating how self-assemblages such as ant rafts are affected by colony size would be of interest [18]. Moreover, a careful investigation of individual behavior as the raft forms would provide novel perspectives on how ants self-organize to form complex structures.

Ants from at least two phylogenetically independent species, *F. selysi* and *S. invicta*, use brood items as a floating platform when they raft. Brood placement in rafts is one of the few examples of hymenopteran societies actively exploiting the functional characteristics of their young, which are usually dependent on adults and only passively contribute to the colony, due to the complete metamorphosis of holometabolous insects. Other examples include weaver ants using silk produced by larvae to build sturdy nests [19], *Leptanilla japonica* brood providing nutrition to queens through a larval hemolymph tap [20] and various forms of brood cannibalism [21], [22].

Overall, collective structures keep nest-mates together during emergencies. Within this function, groups can optimize the structural geometry, taking advantage of the properties of different group members to minimize costs and maximize survival probability. Rafting ants seem to solve this optimization problem by placing brood on the base of the raft, thereby maintaining the colony integrity and constructing a more durable raft without imposing high costs on the brood.

## Supporting Information

File S1 **Contains the files: Text S1** Description of the study species, *Formica selysi.* **Text S2** Additional information about the experimental set up and rafting apparatus. **Text S3** Information on rafting pilot studies. **Text S4** Details about experimental methods. **Text S5** Overview of results from the buoyancy experiment. **Text S6** List of references cited in the supplementary information. **Figure S1** Photo of erosion of *Formica selysi* habitat caused by a flood of the Rhône River. **Figure S2** Side view of the experimental set up. **Table S1** Results of buoyancy tests of workers, brood, and wood cylinders.
(DOC)

Movie S1 60 workers with ten sexual larvae forming a raft, filmed from below and played at 64x speed. Workers place sexual larvae on the base of the raft. We replicated this raft configuration five times with similar results, but provided five sexual pupae instead of the ten larvae shown here to ensure that brood placement was not due solely to the workers’ limited ability to manipulate these large brood items.
(WMV)

Movie S2 60 workers with ten worker brood and ten wood cylinders, played at 64x speed. Workers place brood in a pile as the water level rises and form the raft above the brood. Wood cylinders are not actively collected, but some are incorporated around the perimeter of the raft after the group is afloat.
(WMV)
