# Supplementary material for: Ant Brood Function as Life Preservers during Floods
Source: PLoS One. 2014 Feb 19;9(2):e89211. doi: 10.1371/journal.pone.0089211 (PMC3929613; doi:10.1371/journal.pone.0089211)
Supplement: File S1 — Contains the files: Text S1 Description of the study species, Formica selysi. Text S2 Additional information about the experimental set up and rafting apparatus. Text S3 Information on rafting pilot studies. Text S4 Details about experimental methods. Text S5 Overview of results from the buoyancy experiment. Text S6 List of references cited in the supplementary information. Figure S1 Photo of erosion of Formica selysi habitat caused by a flood of the Rhône River. Figure S2 Side view of the experimental set up. Table S1 Results of buoyancy tests of workers, brood, and wood cylinders. (DOC) [file pone.0089211.s001.doc]

**Supporting Information**

*Text S1. Description of* Formica selysi

Our study species, *Formica selysi,* is abundant in alluvial plains in the mountains of Western Europe, and this habitat is subject to periodic flooding (Fig. S1). Within our study area along the Rhône valley in Valais, Switzerland, we have placed flat stones over colony entrances to facilitate sampling and observation. This population is a mix of single- and multiple-queen (monogynous and polygynous) colonies, and the social structure of each colony has been assessed through parentage analysis using eight microsatellite markers . Mature monogynous colonies from this population contain an average of 3000 workers, while mature polygynous colonies are comprised of an average of 30,000 workers . On average, colonies that produce sexual offspring contain about 200 brood items in a given year , and colonies may contain hundreds up to several thousand worker-destined brood items at one time (pers. obs.).


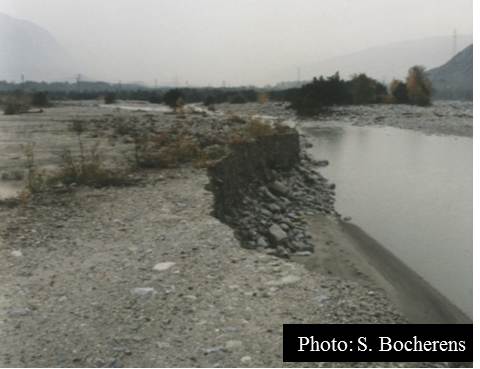


Figure S1: Photo of erosion of *Formica selysi* habitat caused by a flood of the Rhône River.

We used a relatively small number of individuals in our experiments to ensure that we could observe the movements and placement of individuals clearly during the raft assembly process. Field colonies differ in their worker to brood ratio, but the ratios that we use (six workers to one worker-brood item, or 12 workers to one sexual-brood item) are in line with the ratios that we have observed in field colonies.

*Text S2. Experimental set up*

We constructed an apparatus to film raft formation from above and from below simultaneously (Fig. S2). The group of ants was placed on a glass platform consisting of a watch glass (10 cm diameter) mounted on a small plastic container inside a large (38 x 22 x 14 cm), fluon-lined box. This large box contained 3 liters of water surrounding the glass platform. Ants were introduced onto the platform and left to acclimate for at least 5 minutes. For each experiment, we added 1 liter per hour using modified intravenous fluid delivery systems, which allowed for precise calibration of water level rise. We placed portable webcams (Logitech C905) 10 cm both above and below the surface of the watch glass. We began filming 1.5 hours after we initiated water level rise, when the water level reached the edges of the glass platform. We stopped the intravenous drip when the raft was fully formed and had lost contact with the glass surface. We then continued to film after the formation of the ant raft (the duration of rafting varied depending upon the experiment).


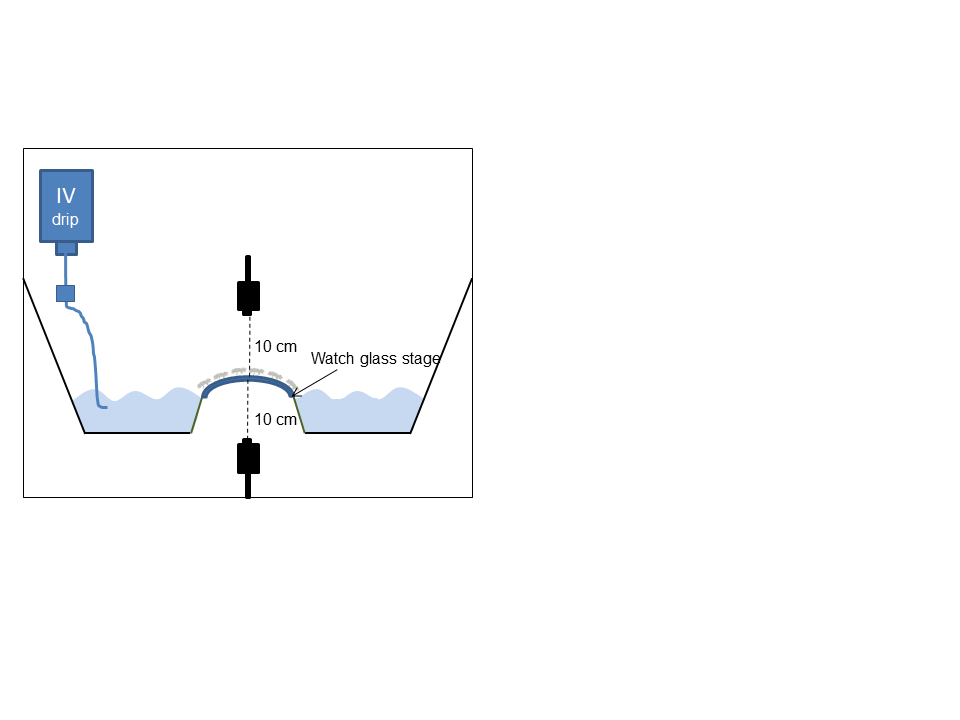


Figure S2: Side view of the experimental set up.

Workers were counted out and placed in a small holding box lined with fluon shortly before rafting. This box was then overturned on the rafting platform to transfer the ants. We placed ants on the experimental platform with water already present in the surrounding box to prevent escape. Initially, the workers were highly dispersed and active, with some entering the water around the platform. These were returned to the platform with soft forceps, and this behavior generally ceased within 5 minutes of transfer. After the workers settled, we placed queens and brood items on the platform one by one using soft forceps. We placed queens and brood away from the largest aggregation of workers, in order to ensure that we would observe how workers would handle them.

*Text S3. Pilot studies*

During the course of these experiments, we tested several additional conditions. Initially, we attempted to induce rafting behavior by submerging colonies kept in a standard large box with sand, but no experimental platform. Under these conditions, ants tended to burrow into the sand. In the field, many *F. selysi* colonies have been observed to remain in submerged ground during floods (Lude et al. 1999), and this behavior may be the first response of a colony to an emergency, particularly if the water level rises suddenly. In contrast, fire ants will quickly form rafts and under a variety of conditions (Mlot et al. 2011). From these observations, we conclude that *F. selysi* is more reluctant to raft than *S. invicta.*

We initiated tests of brood placement in the early spring, when only large sexual brood were present in field colonies. At first, we placed ten sexual larvae in groups of 60 workers. Under these conditions, workers still floated on top of sexual larvae, but the larvae were large relative to the workers, and the raft contained only two layers: the sexual brood on the base and the workers holding the brood from above and maintaining raft cohesion through tarsus-tarsus connections with each other on the top. In order to distinguish between passive and active brood placement, we reduced the number of brood items, in an effort to ensure that workers could physically place brood at interior positions in the colony.

*Text S4. Detailed experimental methods*

*Worker submersion tolerance*

We placed three ants from each of 14 colonies (seven polygynous, seven monogynous) in individual tubes filled with water, which were themselves submerged in water, so that the ants were unable to float to the surface. The ants were left under water for eight hours. We then removed the workers and placed them individually on filter paper. We observed the time elapsed until individuals first began to move and then to walk. We rechecked the workers 24 hours after the end of the submersion experiment to assess the proportion that survived.

*Worker and brood buoyancy*

Mlot et al. demonstrated that the hydrophobic cuticle of fire ants allows them to float in pure water by capturing a pocket of air around their bodies, but that they lose this property, and thus their buoyancy, at low detergent concentrations. To investigate whether *F. selysi* workers and brood items of different instars differ in buoyancy, we placed adult workers, larvae, pupae with cocoon and pupae without cocoon from the same colony of origin in beakers containing distilled water, 0.5% Tween 20 solution, or 1% Tween 20 solution. For each detergent concentration, we used eight replicates per category of worker and brood (sampling one individual from each of four polygynous and four monogynous colonies, respectively). We placed individuals on the surface of the liquid in the center of the beaker and recorded whether they were floating after two minutes. We used a Fisher’s exact test to compare the proportion of workers and brood that remained floating in each solution.

*Raft recovery*

We investigated whether rafts containing brood exhibited a higher survival rate and more rapid recovery after rafting than rafts consisting of workers alone. We collected workers and brood from each of 10 field colonies, and for each colony formed two experimental rafting groups, one containing 60 workers with 10 brood items (larvae and pupae), and the other 60 workers without brood. These groups were the same as the ones used for the rafting tolerance of brood experiment. We let the groups raft for three hours before removing them to dry plastic boxes (22 x 16 x 14 cm) containing a layer of absorbent paper and a plaster nest. We filmed the post-rafting behavior of ants for one hour and measured two factors demonstrating the ability of a group to recover from rafting: the time to disassemble the raft (until workers were no longer joined together by tarsus or mandible) and the number of unresponsive workers. We used paired t-tests to compare the time to disassemble the raft and the number of immobile workers after rafting with or without brood.

After the raft recovery session, workers and brood were placed in boxes (15 x 13 x 6 cm) containing one plaster nest and *ad libitum* access to standard ant food and water. Ten brood items from the same colony of origin were placed with the workers that rafted without brood. These brood items were from the same cohort and were collected on the same day as those used in the rafting trial. We monitored brood survival at least five times per week until all brood eclosed.

*Text S5. Buoyancy results*

All brood items and wood cylinders used in the buoyant materials choice experiment were highly buoyant, even in detergent solution, whereas workers only remained afloat in distilled water (Table S1).

Table S1: Results of buoyancy tests of workers, brood, and wood cylinders.

|  |  | *Proportion afloat* | | |  |
| --- | --- | --- | --- | --- | --- |
| individual | N | distilled water | 0.05% Tween detergent solution | 1% Tween detergent solution | comparison |
| Workers | 1 individual from each of 8 colonies in each solution | 0.875 | 0 | 0 | Buoyancy of workers compared to brood: Fisher's exact test p = 0.012 |
| Larvae | 8 in each | 1 | 1 | 0.875 |
| Pupae with cocoon | 8 in each | 1 | 1 | 0.875 |
| Pupae without cocoon | 8 in each | 1 | 1 | 1 |
| Wood cylinders | 10 in each | 1 | 1 | 1 |  |

*Text S6. References*

Mlot NJ, Tovey CA, Hu DL, 2011. Fire ants self-assemble into waterproof rafts to survive floods. Proceedings of the National Academy of Sciences of the United States of America 108:7669-7673.

Purcell J, Chapuisat M, 2013. Bidirectional shifts in colony queen number in a socially polymorphic ant population. . Evolution 67:1169-1180.

Rosset H, Chapuisat M, 2006. Sex allocation conflict in ants: when the queen rules. Current Biology 16:328-331.

Rosset H, Chapuisat M, 2007. Alternative life-histories in a socially polymorphic ant. Evol Ecol 21:577-588.
